# German oncology certification system for colorectal cancer – relative survival rates of a single certified centre vs. national and international registry data

**DOI:** 10.1515/iss-2021-0002

**Published:** 2021-04-30

**Authors:** Maximilian Richter, Lena Sonnow, Amir Mehdizadeh-Shrifi, Axel Richter, Rainer Koch, Alexander Zipprich

**Affiliations:** Practice Centre Rethen, Centre for General Medicine, Academic Teaching Practice of Hannover Medical School, Hannover, Germany; Department of Diagnostic and Interventional Radiology, Hannover Medical School, Hannover, Germany; Department of General Surgery, University Hospital Basel, Basel, Switzerland; Department of General Surgery, Hospital Hildesheim, Academic Teaching Hospital of Hannover Medical School, Hannover, Germany; Department of Medical Statistics and Biometry, Medical Faculty Carl Gustav Carus at Technical University Dresden, Dresden, Germany; Department for Internal Medicine I, University Hospital Halle/Saale, Halle/Saale, Germany

**Keywords:** cancer registry, certification system, colorectal cancer, epidemiology, oncology

## Abstract

**Objectives:**

To evaluate how the certification of specialised Oncology Centres in Germany affects the relative survival of patients with colorectal cancer (CRC) by means of national and international comparison.

**Methods:**

Between 2007 and 2013, 675 patients with colorectal cancer, treated at the Hildesheim Hospital, an academic teaching hospital of the Hannover Medical School (MHH), were included. A follow-up of the entire patient group was performed until 2014. To obtain international data, a SEER-database search was done. The relative survival of 148,957 patients was compared to our data after 12, 36 and 60 months. For national survival data, we compared our rates with 41,988 patients of the Munich Cancer Registry (MCR).

**Results:**

Relative survival at our institution tends to be higher in advanced tumour stages compared to national and international cancer registry data. Nationally we found only little variation in survival rates for low stages CRC (UICC I and II), colon, and rectal cancer. There were notable variations regarding relative survival rates for advanced CRC tumour stages (UICC IV). These variations were even more distinct for rectal cancer after 12, 36 and 60 months (Hildesheim Hospital: 89.9, 40.3, 30.1%; Munich Cancer Registry (MCR): 65.4, 28.7, 16.6%). The international comparison of CRC showed significantly higher relative survival rates for patients with advanced tumour stages after 12 months at our institution (77 vs. 54.9% for UICC IV; raw p<0.001).

**Conclusions:**

Our findings suggest that patients with advanced tumour stages of CRC and especially rectal cancer benefit most from a multidisciplinary and guidelines-oriented treatment at Certified Oncology Centres. For a better evaluation of cancer treatment and improved national and international comparison, the creation of a centralised national cancer registry is necessary.

## Introduction

Colorectal cancer (CRC) is the third most common cancer in men and the second in women worldwide. Between one and two million cases are diagnosed every year [1]. Furthermore, it is also one of the leading causes for cancer-related deaths worldwide alongside lung cancer and breast cancer [2], [3]. In Germany, the 5-year-prevalence for CRC (ICD-10, C18-21) was 116,000 among men and 98,000 among women in 2013 [4]. Moreover, there was an increase of 38% in the 5-year-prevalence rate for women and 79% in men between 1990 and 2004 in Germany [4]. The German Robert Koch Institute (RKI) estimated the age-standardised incidence rate of CRC at approximately 63,000 cases in 2014; 35,000 among men and 28,000 among women [4]. Therefore, a more integrative and multidisciplinary approach with optimised clinical pathways is necessary to successfully handle the rising numbers of CRC. There are various studies suggesting that patients may benefit from cancer treatment in specialised and centralised institutions, which are often referred to as high-volume hospitals [5], [6], [7], [8], [9]. Taking the above mentioned into consideration, the German government implemented political measures to support the development of centralised cancer care. In 2008, the national cancer plan was initiated by German cancer societies including the German Cancer Society (DKG) and the Federal Ministery of Health (BMG). Four goals were set: reducing cancer-specific mortality through screening programmes, treatment decisions should be made according to evidence-based guidelines in order to maintain quality assurance, efficient oncological treatment should be associated with the collaboration of federal cancer registries, increased patient orientation should improve the quality of oncological care [10]. We aimed to analyse the importance of the German certification system and its benefits for long-term patient survival. We also intended to solidify the necessity for integrative patient treatment and a multidisciplinary centralised approach for CRC.

## Methods and materials

### Study design

The study protocol was presented to the ethics committee of the Medical Association of Lower Saxony and successfully underwent the official processing. Informed consent and assent for prospective data collection were officially obtained from all patients. The outcome assessment was performed retrospectively.

### Subjects

Between 2007 and 2013, we recruited 675 patients (388=57% men; 287=43% women; median age: 71 years) with newly diagnosed CRC at the Hildesheim Hospital. The patients underwent detailed follow-up in 2014.

### Patient data acquisition

The analysed data was requested from the GTDS-database (Gießener Tumordokumentationssystem) which is used for patient data management at the Hildesheim Hospital [11]. The GTDS allows the user to analyse cancer and patient-specific data. Furthermore, detailed information about the individual course of the disease, including survival, are provided by the software [11]. The Hospital is certified by Onkozert as an official Oncology Centre. The hospital also serves as an Academic Teaching Hospital of the Hannover Medical School and has a capacity of 566 beds and 19 specialised departments.

From 2007 to 2013, all documented primary cases of CRC, that underwent elective surgery at the Hildesheim Hospital, were included. Malignancies of the colon, the rectosigmoid transition zone and the rectum (ICD-10, C18-20) were included. Data comprised patients with R0-, R1- and R2-resections. Patients with newly diagnosed malignancies of the anus and the anal canal (ICD-10, C21) were excluded from the study.

### SEER-database

The SEER-database (Surveillance, Epidemiology, and End Results) of the US National Cancer Institute (NCI) contains patient data regarding demographic aspects, tumour localisation, tumour stage, and survival data from different national cancer registries. The database was accessed after online registration at https://seer.cancer.gov/seertrack/data/request/ and downloading the dial-up software (SEER*Stat version 8.2.1; 4/8/2015). The request is based on Incidence – SEER 18 Regs Research Data + Hurricane Katrina Impacted Louisiana Cases, Nov 2015 Sub (1973–2013) data record [12].

All cases of CRC between 2007 and 2013 were included. A request was made for “colon” and “rectum”. Additional search criteria for “sexes”, “all races”, as well as “all ages”, were added. Further filter settings were microscopical confirmation, malignancy, known age, and active follow-up. The total number of analysed patients was 148,957 for CRC, 103,613 for colon cancer and 33,850 for rectal cancer.

Any case with missing data for malignancy, age, follow-up and patients with cancer diagnosed by autopsy or by death certificate were excluded.

### National comparison

For national comparison, we used publicly accessible data from the Munich Cancer Registry (MCR) for the years 1998–2019. The MCR is a clinical cancer registry of the Faculty of Medicine at the University of Munich. Approximately, five million people belong to the catchment area. The data has been provided by cooperating hospitals and practising physicians, as well as health authorities of the neighbouring districts [13], [14], [15], [16].

All registered patients from 1998 to 2019 were included. The total number of evaluated cases was 41,988 for CRC, 26,811 for colon cancer, and 15,177 for rectal cancer.

Patients without information about the above-mentioned characteristics were excluded.

### Statistical analysis

Relative survival was calculated using population-based mortality data from 2007 to 2014 for the region Lower Saxony (Niedersachsen) by a SAS^®^ software-based program (V9.4, SAS Institute, Cary, NC, USA).

The starting point for the overall survival (OAS) was defined as the absence of tumours after curative surgery. The endpoint was the last information available or death. The cut-off date for patient follow-up was November-16-2014 so that a minimum 10-month observation period was ensured. In the case of patients with a loss to follow-up, the data were censored. The graphic display was made by using GraphPad Prism (Version 6.00 for Macintosh, GraphPad Software, La Jolla California USA, www.graphpad.com ). The calculated relative survival after 12, 36, and 60 months was compared with the SEER data and also analysed. Calculating relative survival granted us a direct comparison of the two population groups with regard to different distributions of age, gender, and year of operation. Since the original data of the cancer registries was not analysed, Wald tests were used to allow an approximate comparison of fixed time points when confidence intervals or standard errors were available (SEER data). The Munich Cancer Registry data did not include confidence intervals and standard errors, therefore a simple tabular comparison of relative survival rates was performed.

## Results

From 2007 to 2013, the 5-year relative survival rate for colorectal cancer at the Hildesheim Hospital was 65.3% (95CI; 59.6–71.7%). The rate was calculated for both sexes and without stratification. The result was independent of the underlying surgical method.

**Figure 1:**
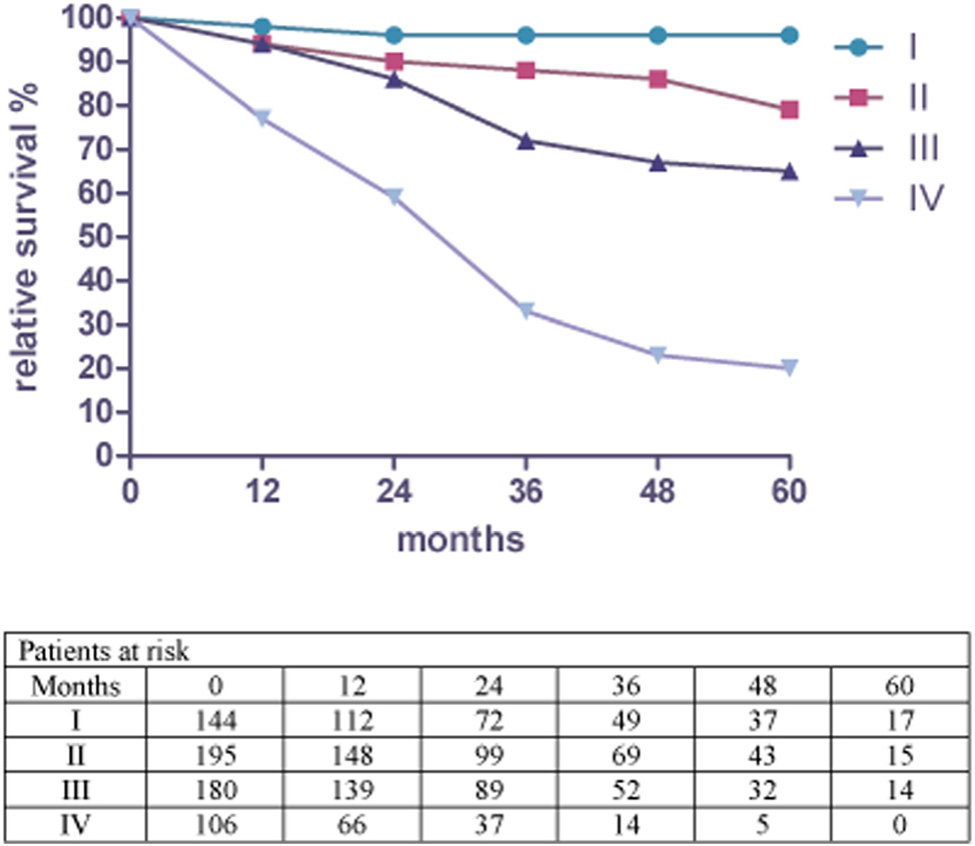
Relative survival by stage for colorectal cancer and patients at risk from 2007 to 2013 in Hildesheim.

Figure 1 shows the relative 5-year survival rate for colorectal cancer from 2007 until 2013 at the Hildesheim Hospital. Relative survival for (p)UICC-stage I was 95.5% (95% CI; 86.6–105%). Stage II and Stage III showed survival rates of 79.4% (95% CI; 68.3–92.4%) and 64.7% (95% CI; 53.8–77.8%), whereas Stage IV was 19.7% (95% CI; 11.3–34.4%).

**Figure 2:**
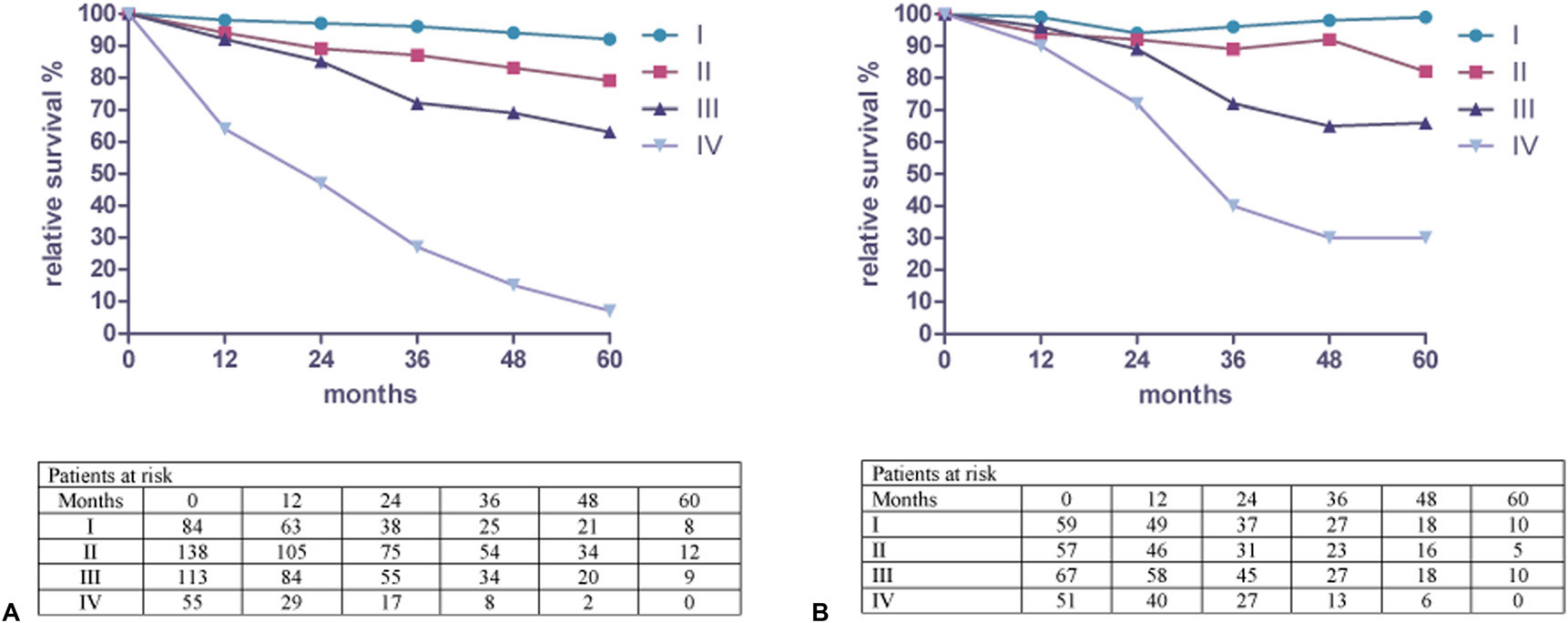
(a) Relative survival by stage for colon cancer and patients at risk from 2007 to 2013 in Hildesheim. (b) Relative survival by stage for rectal cancer and patients at risk from 2007 to 2013 in Hildesheim.

Figure 2a shows the relative 5-year survival rate for colon cancer from 2007 until 2013 at the Hildesheim Hospital. The rates were calculated for both sexes and results were independent of the underlying surgical method. Relative survival for (p)UICC-stage I was 91.9% (95% CI; 77.9–108%). Stage II was 78.7% (95% CI; 65.3–94.9%) and stage III 63.2% (95% CI; 51.5–77.5%). Stage IV showed a survival rate of 7.3% (95% CI; 2.7–19.8%).

Figure 2b shows the relative 5-year survival rate for rectal cancer from 2007 until 2013 at the Hildesheim Hospital. The rates were calculated for both sexes. The results were independent of the underlying surgical method. Relative survival for (p)UICC-stage I was 99.4% (95% CI; 89.9–110%). Stage II was 81.5% (95% CI; 63.8–104%) and stage III 65.6% (95% CI; 51.5–83.5%). Stage IV showed rates of 30.1% (95% CI; 17.8–50.7%).

### National comparison of relative survival

Relative survival for patients with CRC at (p)UICC-stage I after 1, 3, and 5 years were 98, 96.3 and 95.5%, respectively, at the Hildesheim Hospital. Survival at stage II was 93.6, 87.7, 79.4%. Stage III and Stage IV showed survival rates of 93.8, 72.1, 64.7, and 76.8, 33.4, 19.7%. In comparison, the survival rates of the Munich Cancer Registry (MCR) from 1998 to 2019 for patients with CRC at (p)UICC-stage I after 1, 3 and 5 years were 97.4, 97.2 and 95.1%, respectively. Stage II had rates of 93.8, 89.0, 84.0%. Stage III and IV were 91.3, 78.6, 69.1, and 59.2, 25.3, 14.6% (Table 1) [15].

**Table 1:** Relative survival for CRC at the Hildesheim Hospital from 2007 to 2013 compared to registry data of the Munich Cancer Registry from 1998 to 2019.

(p)UICC-stage	12 months	36 months	60 months
I	98% (97.4%)	96.3% (97.2%)	95.5% (95.1%)
II	93.6% (93.8%)	87.7% (89.0%)	79.4% (84.0%)
III	93.8% (91.3%)	72.1% (78.6%)	64.7% (69.1%)
IV	76.8% (59.2%)	33.4% (25.3%)	19.7% (14.6%)

From 2007 to 2013, relative survival at the Hildesheim Hospital for patients with colon cancer at (p)UICC-stage I after 1, 3, and 5 years were 97.5, 96.4 and 91.9%, respectively. Stage II showed rates of 93.6, 86.7, 78.7%. Stage III and IV were 92.4, 72.3, 63.2, 26.9, 7.3%. In comparison, the survival rates of the Munich Cancer Registry (MCR) from 1998 until 2019 for patients with colon cancer at (p)UICC-stage I after 1, 3, and 5 years were 97.4, 97.5 and 96.2%%, respectively. Stage II had rates of 93.7, 89.7, 85.3%. Stage III and IV showed survival rates of 89.9, 76.4, 68.2, and 55.9, 23.4, 13.6% (Table 2) [14].

**Table 2: j_iss-2021-0002_tab_002:** Relative survival colon cancer at the Hildesheim Hospital from 2007 to 2013 compared to registry data of the Munich Cancer Registry from 1998 to 2019.

(p)UICC-stage	12 months	36 months	60 months
I	97.5% (97.4%)	96.4% (97.5%)	91.9% (96,2%)
II	93.6% (93.7%)	86.7% (89.7%)	78.7% (85.3%)
III	92.4% (89.9%)	72.3% (76.4%)	63.2% (68.2%)
IV	64.3% (55.9%)	26.9% (23.4%)	7.3% (13.6%)

From 2007 to 2013, relative survival for patients with rectal cancer at the Hildesheim Hospital at (p)UICC-stage I after 1, 3, and 5 years were 98.6, 95.8 and 99.4%, respectively,. Stage II was 93.5, 89.0, 81.5%. Stage III and IV showed survival rates of 95.5, 72.3, 65.6, and 89.9, 40.3, 30.1%. In comparison, the survival rates of the Munich Cancer Registry (MCR) from 1998 until 2019 for patients with rectal cancer in (p)UICC-stage I after 1, 3, and 5 years were 97.4%, 96.7% and 93.3%, respectively. Stage II had rates of 93.8, 87.6, 80.9%. Stage III and IV showed rates of 93.1, 81.4, 70.3, and 65.4, 28.7, 16.6% (Table 3) [16].

**Table 3:** Relative survival for rectal cancer at the Hildesheim Hospital from 2007 to 2013 compared to registry data of the Munich Cancer Registry from 1998 to 2019.

(p)UICC-stage	12 months	36 months	60 months
I	98.6% (97.4%)	95.8% (96.7%)	99.4% (93.3%)
II	93.5% (93.8%)	89% (87.6%)	81.5% (80.9%)
III	95.5% (93.1%)	72.3% (81.4%)	65.6% (70.3%)
IV	89.9% (65.4%)	40.3% (28.7%)	30.1% (16.6%)

In summary, the national comparison between our single institution and the Munich Cancer Registry showed little variations in relative survival for UICC stages I to III (Table 1 [Table j_iss-2021-0002_tab_002]–3). A difference for all entities was observed at UICC stage IV in favour of our Certified Oncology Centre. The most substantial difference was seen in rectal cancer after 12 months with up to 25 percentage points (Table 3).

### International comparison

From 2007 to 2013, relative survival at the Hildesheim Hospital for patients with CRC after 1, 3, and 5 years for stage “local” was 92, 85 and 81%, respectively. For stage “regional”, it was 94, 72 and 65%. Stage “distant” showed a relative survival of 77, 33 and 20%. In comparison, the relative survival of the SEER-data for stage “local” was 95.5, 92.8 and 90.2%. For stage “regional”, it was 87.6, 71.1 and 60.5%. Stage “distant” showed a relative survival of 54.9, 23.6 and 13.7% (Table 4) [12].

**Table 4:** International comparison of relative survival data for colorectal cancer from 2007 to 2013.

SEER-stage/	Relative survival	Relative survival	p-Values
UICC-stage/	% SEER	% Hildesheim	(Raw)
Months	2007–2013	2007–2013	
Local, I + II, 12	95.5	92	0.0434
Local, I + II, 36	92.8	85	0.0037
Local, I + II, 60	90.2	81	0.0170
Regional, III, 12	87.6	94	0.0560
Regional, III, 36	71.1	72	0.8461
Regional, III, 60	60.5	65	0.4636
Distant, IV, 12	54.9	77	<0.001
Distant, IV, 36	23.6	33	0.0825
Distant, IV, 60	13.7	20	0.2834

From 2007 to 2013, the relative survival for colon cancer at the Hildesheim Hospital after 1, 3, and 5 years were 92, 85 and 79%, respectively, for stage “local”. For stage “regional”, it was 92, 72 and 63%. Stage “distant” showed a relative survival of 64, 27 and 7%. In comparison, the relative survival of the SEER-data for the same period was 95.4, 93.3 and 91.1% for stage “local”. For stage “regional”, it was 86, 69.2 and 59.7%. Stage “distant” showed a relative survival of 52.5, 22.4 and 13.5% (Table 5) [12].

**Table 5:** International comparison of relative survival data for colon cancer from 2007 to 2013.

SEER-stage/	Relative survival	Relative survival	p-Values
UICC-stage/	% SEER	% Hildesheim	(Raw)
Months	2007–2013	2007–2013	
Local, I + II, 12	95.4	92	0.1432
Local, I + II, 36	93.3	85	0.0221
Local, I + II, 60	91.1	79	0.0261
Regional, III, 12	86.0	92	0.0587
Regional, III, 36	69.2	72	0.6446
Regional, III, 60	59.7	63	0.6201
Distant, IV, 12	52.5	64	0.093
Distant, IV, 36	22.4	27	0.5373
Distant, IV, 60	13.5	7	0.1369

From 2007 until 2013, the relative survival for patients with rectal cancer at the Hildesheim Hospital after 1, 3, and 5 years were 92, 85 and 84%, respectively, for stage “local”. For stage “regional” it was 96, 72 and 66%. Stage “distant” showed a relative survival of 90, 40 and 30%. In comparison, the relative survival of the SEER-data for the same period was 95.8, 91.7 and 87.9% for stage “local”. For stage “regional” it was 92, 75.4 and 61.8%. Stage “distant” showed a relative survival of 61.9, 26.3 and 13.7% (Table 6) [12].

**Table 6:** International comparison of relative survival data for rectal cancer from 2007 to 2013.

SEER-stage/	Relative survival	Relative survival	p-Values
UICC-stage/	% SEER	% Hildesheim	(Raw)
Months	2007–2013	2007–2013	
Local, I + II, 12	95.8	92	0.1604
Local, I + II, 36	91.7	85	0.0957
Local, I + II, 60	87.9	84	0.4529
Regional, III, 12	92	96	0.2266
Regional, III, 36	75.4	72	0.6337
Regional, III, 60	61.8	66	0.61
Distant, IV, 12	61.9	90	<0.001
Distant, IV, 36	26.3	40	0.083
Distant, IV, 60	13.7	30	0.0526

In summary, the international comparison showed a significantly better relative survival in CRC and colon stages I–II for the SEER registry data, whereas no significant difference was observed for rectal cancer in UICC stages I–II. In contrast, a better relative survival was seen at our institution for advanced tumour stages (UICC III–IV).

After 12 months, a significant difference (raw p<0.001) of more than 20% was seen for CRC (UICC IV) and rectal cancer (UICC IV) at the Certified Oncology Centre (Table 4 and 6).

## Discussion

In this study, we compare relative survival rates of patients with colorectal cancer at a Certified Oncology Centre, accredited by the German Cancer Society (DKG), with national survival rates published by the Munich Cancer Registry (MCR) and international survival rates obtained from the American SEER-database [12], [13].

For early tumour stages, we noticed only minimal variations between the relative survival rates at our institution and the compared national data. In international comparison, there was a significantly better survival at early tumour stages in favour of the SEER data. Both, for national and international data we recognised a trend towards higher survival rates in advanced tumour stages (UICC III–IV) – especially for CRC and rectal cancer at our institution.

To analyse differences in treatment quality we compared the relative survival of a single institution with German registry data from the Munich Cancer Registry (MCR) and the SEER database, comprising data by different types of hospitals and private practices. Including certified as well as non-certified hospitals, we assumed that relative survival at a single certified institution would be better than the compared pooled data.

Significant differences were seen for advanced tumour stages with a relative survival benefit for patients treated at a certified institution as compared to the national and international databases. The success of the initial surgical treatment is crucial for the final outcome of CRC patients and is often more challenging in advanced tumour stages, requiring a high surgical expertise. Since the certification process is accompanied by a minimum number of procedures performed by surgeon, a certain level of experience can be presumed [17]. According to the benchmark report 2013 by OnkoZert [18], the Hildesheim Hospital had 60 primary cases of colon and 32 cases of rectal cancer in 2011. The median of 230 other certified institutions was 50.5 cases (range 25–171) for colon cancer and 26 cases (range 12–106) for rectal cancer. This supports our assumption that Hildesheim Hospital is a representative institution within other certified centres.

A literature review revealed studies that showed a correlation between hospital volume and a better outcome in colorectal surgery [5], [6], [7], [8]. This volume-outcome relationship appears to be even stronger for the individual surgeon than for the hospital [19]. According to the German Cancer Society (DKG) surgeons at a Certified Cancer Centre require 15 colon and 10 rectal surgical procedures per year as a minimum requirement [17]. The American Leapfrog Group sets the minimal hospital volume for rectal surgery at 16 cases per year and six per surgeon [20]. These variations need to be considered when comparing national and international data, a clear definition of the required hospital volume could be useful. As advanced tumour stages of CRC often present as complex diseases, a multidisciplinary treatment approach may appear to be most beneficial in this patient group. We assume that the multidisciplinary and integrative approach, offered at a Certified Oncology Centre, may lead to better patient care and improved overall survival. The multidisciplinary approach as defined by DKG includes parameters like pre-therapeutical and post-operative interdisciplinary case presentations, specific (immuno-)histopathological analyses (e.g. microsatellite instability), psycho-oncological care, social assistance, genetic counselling and participation in medical trials. Depending on the tumour stage systemic therapies are initiated in collaboration with the oncology department. Moreover, the precise documentation of complications and patient follow-up is necessary [18]. The combination of the mentioned factors may have an important influence on survival.

For early-stage CRC we observed better survival rates for the international pooled data. This could be based on the high level of standardisation and increasing expertise in the treatment of patients with early-stage CRC, as well as the implementation of improved guidelines. Hereby the influence of the certification by itself seems to be more substantial for patients with advanced tumour stages and probably with concomitant comorbidities.

The ongoing certification has led to different paradigm changes in the treatment of CRC in the last decade. A study from Kowalski et al. outlined three fundamental changes caused by the implementation of the certification system – multidisciplinarity, fair processing for certification and improved collection of data for comparison [21]. Among these three changes, we believe that multidisciplinary treatment is the most important factor influencing our calculated survival rates. A recent study by Wesselmann et al. supports this assumption and also emphasizes the standardised multidisciplinary treatment. A focus on interdisciplinary collaboration is an important quality indicator in the certification process and a major goal of OnkoZert and the DKG to improve CRC treatment across Germany [22]. Some recent studies could already prove the benefit of the certification process for CRC, e.g. Völkel et al. analysed data of a clinical cancer registry (Tumorzentrum Regensburg) and demonstrated patients having a long-term survival benefit when treated at certified cancer centres compared to non-certified hospitals [23]. Another study by Trautmann et al. indicates that the implementation and assurance of evidence-based quality standards have substantial positive effects on various patient-relevant outcomes in colon cancer care [24].

A major challenge accompanying such studies remains the complexity of the multifactorial evaluation making it difficult to identify all contributing aspects that may interfere with the treatment. Since individual hospital reports often do not include relative survival rates, a national cancer registry may improve the evaluation and the outcome of the certification process. Similarly, meaningful international comparisons are still complicated by the lack of a centralised national cancer registry. Though improvements in transparency and increased implementation of the certification across Germany were achieved, more research needs to be done to better evaluate the impact of the certification on the treatment of colorectal cancer in Germany and worldwide.

Regarding our study, there is a limitation due to its retrospective design and the difference of the registry data. Moreover, no detailed information on additional patient characteristics, such as comorbidities, psycho-oncological factors, and chemoradiotherapy was available.

In summary, we conclude that advanced stages of CRC and rectal cancer benefit most from treatment at a Certified Cancer Centre. We assume the effect being mostly based on the multidisciplinary and integrative approach.

## References

[1] Ferlay J, Soerjomataram I, Dikshit R, Eser S, Mathers C, Rebelo M, . Cancer incidence and mortality worldwide: sources, methods and major patterns in GLOBOCAN 2012. Int J Canc 2015;136:E359–86. 10.1002/ijc.29210 25220842

[2] Bray F, Ferlay J, Soerjomataram I, Siegel RL, Torre LA, Jemal A. Global cancer statistics 2018: GLOBOCAN estimates of incidence and mortality worldwide for 36 cancers in 185 countries. CA Cancer J Clin 2018;68:394–424. 30207593 10.3322/caac.21492

[3] Erratum. Global cancer statistics 2018: GLOBOCAN estimates of incidence and mortality worldwide for 36 cancers in 185 countries. CA Cancer J Clin 2020;70:313 313. 10.3322/caac.21609 32767693

[4] Bertz J, Dahm S, Haberland J, Kraywinkel K, Kurth B-M, Wolf U. Verbreitung von Krebserkrankungen in Deutschland [Internet], Available from https://edoc.rki.de/handle/176904/3226 [Accessed 3 Jan 2021]..

[5] El Amrani M, Clement G, Lenne X, Rogosnitzky M, Theis D, Pruvot F-R, . The impact of hospital volume and Charlson score on postoperative mortality of proctectomy for rectal cancer: a nationwide study of 45,569 patients. Ann Surg 2018;268:854–60. 30063493 10.1097/SLA.0000000000002898

[6] Pucciarelli S, Zorzi M, Gennaro N, Marchegiani F, Barina A, Rugge M, . Relationship between hospital volume and short-term outcomes: a nationwide population-based study including 75,280 rectal cancer surgical procedures. Oncotarget 2018;9:17149–59. 29682212 10.18632/oncotarget.24699 PMC5908313

[7] Aquina CT, Probst CP, Becerra AZ, Iannuzzi JC, Kelly KN, Hensley BJ, . High volume improves outcomes: the argument for centralization of rectal cancer surgery. Surgery 2016;159:736–48. 26576696 10.1016/j.surg.2015.09.021

[8] Huo YR, Phan K, Morris DL, Liauw W. Systematic review and a meta-analysis of hospital and surgeon volume/outcome relationships in colorectal cancer surgery. J Gastrointest Oncol 2017;8:534–46. 28736640 10.21037/jgo.2017.01.25 PMC5506277

[9] Birkmeyer JD, Siewers AE, Finlayson EVA, Stukel TA, Lucas FL, Batista I, . Hospital volume and surgical mortality in the United States. N Engl J Med 2002;346:1128–37. 11948273 10.1056/NEJMsa012337

[10] Broschuere_Nationaler_Krebsplan.pdf [Internet]. Federal Ministry of Health Available from: https://www.bundesgesundheitsministerium.de/fileadmin/Dateien/5_Publikationen/Praevention/Broschueren/Broschuere_Nationaler_Krebsplan.pdf [Accessed 3 Jan 2021]..

[11] Altmann U, Katz FR, Tafazzoli AG, Haeberlin V, Dudeck J. GTDS--a tool for tumor registries to support shared patient care. Proc – Conf Am Med Inf Assoc Annu Fall Symp 1996:512–6. PMC2232993 8947719

[12] About the SEER program [Internet], Available from https://seer.cancer.gov/about/overview.html [Accessed 3 Jan 2021]..

[13] Munich Cancer Registry (MCR). Catchment area of MCR [Internet], Available from https://www.tumorregister-muenchen.de/en/area.php [Accessed 3 Jan 2021]..

[14] sC18__E-ICD-10-C18-colon-cancer-survival.pdf [Internet], Available from https://www.tumorregister-muenchen.de/en/facts/surv/sC18__E-ICD-10-C18-Colon-cancer-survival.pdf [Accessed 24 Feb 2021]..

[15] sC1820E-ICD-10-C18-C20-colorectal-cancer-survival.pdf [Internet], Available from https://www.tumorregister-muenchen.de/en/facts/surv/sC1820E-ICD-10-C18-C20-Colorectal-cancer-survival.pdf [Accessed 24 Feb 2021]..

[16] sC1920E-ICD-10-C19-C20-rectal-cancer-survival.pdf [Internet], Available from https://www.tumorregister-muenchen.de/en/facts/surv/sC1920E-ICD-10-C19-C20-Rectal-cancer-survival.pdf [Accessed 24 Feb 2021]..

[17] DKG. Zertifizierung der Deutschen Krebsgesellschaft: Dokumente [Internet], Available from https://www.krebsgesellschaft.de/zertdokumente.html [Accessed 3 Jan 2021]..

[18] Individual Benchmark Report of Intestinal Cancer Centers, German Cancer Society (DKG). Key figures and indicators analysis. Darmzentrum Hildesheim; 2013 FAD-Z037.

[19] Archampong D, Borowski D, Wille‐Jørgensen P, Iversen LH. Workload and surgeon’s specialty for outcome after colorectal cancer surgery, Available from https://www.cochranelibrary.com/cdsr/doi/10.1002/14651858.CD005391.pub3/full [Accessed 3 Jan 2021].. 10.1002/14651858.CD005391.pub3 PMC12076000 22419309

[20] 2020 surgical volume-appropriateness fact sheet.pdf [Internet], Available from https://ratings.leapfroggroup.org/sites/default/files/inline-files/2020%20Surgical%20Volume-Appropriateness%20Fact%20Sheet.pdf [Accessed 3 Jan 2021]..

[21] Kowalski C, Graeven U, von Kalle C, Lang H, Beckmann MW, Blohmer J-U, . Shifting cancer care towards multidisciplinarity: the cancer center certification program of the German Cancer Society. BMC Canc 2017;17:850. 10.1186/s12885-017-3824-1 PMC5731059 29241445

[22] Wesselmann S, Winter A, Ferencz J, Seufferlein T, Post S. Documented quality of care in certified colorectal cancer centers in Germany: German Cancer Society benchmarking report for 2013. Int J Colorectal Dis 2014;29:511–8. 24584335 10.1007/s00384-014-1842-x

[23] Völkel V, Draeger T, Gerken M, Fürst A, Klinkhammer-Schalke M. Long-term survival of patients with colon and rectum carcinomas: is there a difference between cancer centers and non-certified hospitals?. Gesundheitswesen Bundesverb Arzte Offentl Gesundheitsdienstes Ger 2019;81:801–7. 10.1055/a-0591-3827 29672814

[24] Trautmann F, Reißfelder C, Pecqueux M, Weitz J, Schmitt J. Evidence-based quality standards improve prognosis in colon cancer care. Eur J Surg Oncol 2018;44:1324–30. 29885983 10.1016/j.ejso.2018.05.013

