# Supplementary material for: German oncology certification system for colorectal cancer – relative survival rates of a single certified centre vs. national and international registry data
Source: Innov Surg Sci. 2021 Apr 30;6(2):67–73. doi: 10.1515/iss-2021-0002 (PMC8435270; doi:10.1515/iss-2021-0002)
Supplement: Supplementary file 1 [file iss-06-20210002-s001.pdf]

Reviewer Assessment

L. Sonnow et al.: German oncology certification system for colorectal cancer - a national and international comparison of relative survival rates

Reviewers' Comments to Original Submission

Reviewer 1: Weimann, Arved

Date received: 25-Jan-2021
Reviewer recommendation: **Return to author for major modifications**
Reviewer overall scoring: **Medium**

Assessment Form scores: 5 = High/Yes; 3 = Medium/Adequate; 1 = Low

Is the subject area appropriate for the journal	5				
Does the title clearly reflect the paper's content?			3		
Does the abstract clearly reflect the paper's content		4			
Do the keywords clearly reflect the paper's content?			3		
Does the introduction present the problem clearly?		4			
Are the results/ conclusions justified?			3		
How comprehensive and up-to-date is the subject matter presented?		4			
How adequate is the data presentation?			3		
Are units and terminology used correctly?		4			
Is the number of cases adequate?		4			
Are the experimental methods/ clinical studies adequate?		4			
Is the length appropriate in relation to the content?		4			
Does the reader get new insights from the article?			3		
Please rate the practical significance.			3		
Please rate the accuracy of methods.		4			
Please rate the statistical evaluation and quality control.			3		
Please rate the appropriateness of the figures and tables.		4			
Please rate the appropriateness of the references.		4			
Please evaluate the writing style and use of language.			3		
Please judge the overall scientific quality of the manuscript.		4			
Are the methods used worthy of reproduction in greater deal?					
Would you be willing to review a revision of this manuscript?	Yes				

Comments to author: The authors address a controversially discussed issue regarding the quality of colorectal cancer in certified multidisciplinary centres.

The approach to compare and discuss single-center data with those from accessible national and international registries is innovative.
The statistical analysis does not seem to be simple. Did the authors have statistical advice? How did the authors deal when confidence intervals and standard errors were not available? Please give more details.
It may be discussed whether the results of a single center may represent the general quality of treatment in certified Oncology Centres in Germany. The presented results are excellent but can also just demonstrate the quality of treatment in Hildesheim. It cannot necessarily be related to the certification per se. Ideally, the authors would also provide the corresponding data from Onkozert.
-Please describe the multidisciplinary treatment in Hildesheim more in detail
-Is there information available for the Munich Cancer Registry regarding data from certified and non-certified centres.
-Please discuss the significant difference in the international comparison of colorectal cancer stage I and II.

Reviewer 2: Hiller, Wolfgang

Date received: 30-Jan-2021
Reviewer recommendation: **Return to author for major modifications**
Reviewer overall scoring: **High**

Assessment Form scores: 5 = High/Yes; 3 = Medium/Adequate; 1 = Low

Is the subject area appropriate for the journal		4			
Does the title clearly reflect the paper's content?				2	
Does the abstract clearly reflect the paper's content		4			
Do the keywords clearly reflect the paper's content?			3		
Does the introduction present the problem clearly?			3		
Are the results/ conclusions justified?				2	
How comprehensive and up-to-date is the subject matter presented?			3		
How adequate is the data presentation?		4			
Are units and terminology used correctly?		4			
Is the number of cases adequate?	5				
Are the experimental methods/ clinical studies adequate?		4			
Is the length appropriate in relation to the content?		4			
Does the reader get new insights from the article?				2	
Please rate the practical significance.		4			
Please rate the accuracy of methods.			3		
Please rate the statistical evaluation and quality control.			3		
Please rate the appropriateness of the figures and tables.			3		
Please rate the appropriateness of the references.			3		
Please evaluate the writing style and use of language.		4			
Please judge the overall scientific quality of the manuscript.				2	
Are the methods used worthy of reproduction in greater deal?	No				
Would you be willing to review a revision of this manuscript?	Yes				

Comments to author: The paper addresses an interesting topic of clinical relevance.

The title does not reflect the contents/objective of the study as it compares single center results with national/international cohort studies. To really evaluate the impact of a certification it would be necessary to compare the results of a cohort of certified centers with those of the registry data. The paper still is a study worth publishing.

However, there are some major mistakes and a thorough revision is necessary.

The drop-out rate should be mentioned aswell as the reasons. What happened to patients undergoing R1/R2-resections, especially in UICC stage IV patients?

The data show significantly poorer survival rates for early cancer as compared to the registry (whereas the survival benefit in metastatic disease is not statistically significant). This needs some kind of explanation in the discussion.

There is a severe mix-up with the data:

Lines 137-140 refer to colon cancer and not colorectal; vice versa lines 145-148 reflect the colo-rectal data and not the data of colonic carcinoma.

Table 1 shows the data of colon cancer and not the whole colo-rectal population, whereas table 2 shows the colo-rectal population and not colon carcinoma only. It remains unclear as to whether the reference numbers are valid or the titles of the tables just have been swapped. In the first case, the statistical analysis has to be reevaluated.

References should be cited in English according to international standards.

Authors' Response to Reviewer Comments

Date received: 24-Feb-2021

Response to reviewer 1

The authors address a controversially discussed issue regarding the quality of colorectal cancer in certified multidisciplinary centres.

The approach to compare and discuss single-center data with those from accessible national and international registries is innovative.

The statistical analysis does not seem to be simple. Did the authors have statistical advice?

> Statistical advice was given by Prof. Rainer Koch (em.). Medical Faculty Carl Gustav Carus, Technical University Dresden.

How did the authors deal when confidence intervals and standard errors were not available? Please give more details.

> Standards errors and confidence intervals were available for the raw SEER-data. The estimation error has been recalculated from the raw material. This enabled us to apply *Wald tests* allowing an approximate comparison on fixed time points.

For the Munich Cancer Registry data no confidence intervals and standard errors were available, unfortunately even after request no detailed data were provided. Therefore, we were limited to a simple tabular comparison of the publicly accessible relative survival rates by stage. A more detailed information was added to the manuscript's methods (lines 118-120).

It may be discussed whether the results of a single center may represent the general quality of treatment in certified Oncology Centres in Germany. The presented results are excellent but can also just demonstrate the quality of treatment in Hildesheim. It cannot necessarily be related to the certification per se. Ideally, the authors would also provide the corresponding data from Onkozert.

This is a very reasonable comment. We fully agree that it is difficult to prove an effect of certification itself and to distinguish precisely from other interfering factors. We tried to address this problem by following your advice and supplemented the study with OnkoZert data to better classify the observed hospital within other certified centres. We assume that Hildesheim Hospital is a representative certified single centre regarding important certification parameters e.g. the number of primary cases and the complete resection rate. The information is provided in the manuscript's discussion (lines 223 -228).

Please describe the multidisciplinary treatment in Hildesheim more in detail

To the best of our knowledge there is no special treatment or exclusive multidisciplinary approach offered at the hospital compared to other similar institutions. We added a more detailed description of the usual multidisciplinary management in certified cancer centres as required by Onkozert to the manuscript (lines 241-249).

Is there information available for the Munich Cancer Registry regarding data from certified and non-certified centres.

To the best of our knowledge the only publicly available data dealing with this topic is the final evaluation report of the Munich Cancer Registry of 2012 (Schlesinger-Raab A, Schrodi S, Liebetruth E, Meyer R, Schubert-Fritschle G, Engel J. Versorgungsforschung: Kolorektales Karzinom). In this report no significant influence of the centre status on survival could be proven. A further analysis with larger follow-up intervals was suggested.

Please discuss the significant difference in the international comparison of colorectal cancer stage I and II.

We found a significant difference in early stage CRC in favor of the pooled SEER data. As an explanation we think that comorbidities could be a potential confounder as they are not considered in this kind of registry data. It is likely that patients undergoing elective surgery at a certified cancer centre present themselves with more comorbidities compared to smaller non-certified hospitals. Another reason could be a more elaborated level of standardization in the United States. We added this point to the discussion (lines 250-255).

Response to reviewer 2

The title does not reflect the contents/objective of the study as it compares single center results with national/international cohort studies.

The title was changed from

"German oncology certification system for colorectal cancer - a national and international comparison of relative survival rates"

into

“German oncology certification system for colorectal cancer – relative survival rates of a single certified centre *vs.* national and international registry data”

To really evaluate the impact of a certification it would be necessary to compare the results of a cohort of certified centers with those of the registry data. The paper still is a study worth publishing. However, there are some major mistakes and a thorough revision is necessary.

The drop-out rate should be mentioned as well as the reasons.

Initially there was no drop-out rate calculated for this study. The underlying statistical survival analysis uses censored data. The time point of censoring was the *last available information* of the patient. *The last information available* could be any contact of the patient with the hospital, stationary or as outpatient treatment, therefore allowing us to classify the patient as living on that specific time point. A routinely performed follow-up questionnaire was sent to the patients by mail. Patients who were lost to follow up were treated as censored and therefore included in the study. We added this information to the manuscript (lines 109-110).

What happened to patients undergoing R1/R2-resections, especially in UICC stage IV patients?

We included all primary cases of CRC between 2007 and 2013 regardless of the resection status. R0, R1, R2 as well as missing information was taken into account. We added this information to the manuscript (lines 73-74).

The data show significantly poorer survival rates for early cancer as compared to the registry (whereas the survival benefit in metastatic disease is not statistically significant). This needs some kind of explanation in the discussion.

We changed the results and some discussion sections in order to address your mentioned point appropriately (lines 193-197, 205-210, 250-255).

The observed survival benefit in stage IV CRC and rectal cancer in international comparison is statistically significant after one year whereas significance diminishes after three and five years. This could be attributed to the low case number in this group or just reflect the natural course of the disease. Given the palliative situation and low life expectancy in this setting the advantage of survival after one year must be evaluated as highly relevant.

There is a severe mix-up with the data:

Lines 137-140 refer to colon cancer and not colorectal; vice versa lines 145-148 reflect the colo-rectal data and not the data of colonic carcinoma.

Table 1 shows the data of colon cancer and not the whole colo-rectal population, whereas table 2 shows the colo-rectal population and not colon carcinoma only. It remains unclear as to whether the reference numbers are valid or the titles of the tables just have been swapped. In the first case, the statistical analysis has to be reevaluated.

Thank you very much for the very important advice. We apologize for any inconvenience regarding the confusion. We thoroughly reanalysed our raw data and changed the data in table one and two as well as the corresponding results section (lines 143-154). We also updated the data of the Munich Cancer registry up to the year 2019.

References should be cited in English according to international standards.

Thank you again for your comment. We changed the references accordingly.

Reviewers' Comments to Revised Submission

Reviewer 1: Weimann, Arved

Date received: 10-Mar-2021
Reviewer recommendation: **Accept in present form**
Reviewer overall scoring: **High**

Assessment Form scores: 5 = High/Yes; 3 = Medium/Adequate; 1 = Low

Is the subject area appropriate for the journal		4			
Does the title clearly reflect the paper's content?		4			
Does the abstract clearly reflect the paper's content		4			
Do the keywords clearly reflect the paper's content?		4			
Does the introduction present the problem clearly?		4			
Are the results/ conclusions justified?			3		
How comprehensive and up-to-date is the subject matter presented?		4			
How adequate is the data presentation?		4			
Are units and terminology used correctly?		4			
Is the number of cases adequate?		4			
Are the experimental methods/ clinical studies adequate?		4			
Is the length appropriate in relation to the content?		4			
Does the reader get new insights from the article?			3		
Please rate the practical significance.			3		
Please rate the accuracy of methods.		4			
Please rate the statistical evaluation and quality control.		4			
Please rate the appropriateness of the figures and tables.		4			
Please rate the appropriateness of the references.		4			
Please evaluate the writing style and use of language.			3		
Please judge the overall scientific quality of the manuscript.		4			

Are the methods used worthy of reproduction in greater deal?	Yes
Would you be willing to review a revision of this manuscript?	

Comments to author: No further comments.

Reviewer 2: Hiller, Wolfgang

Date received: 14-Mar-2021
Reviewer recommendation: **Accept in present form**
Reviewer overall scoring: **Medium**

Assessment Form scores: 5 = High/Yes; 3 = Medium/Adequate; 1 = Low

Is the subject area appropriate for the journal		4			
Does the title clearly reflect the paper's content?		4			
Does the abstract clearly reflect the paper's content		4			
Do the keywords clearly reflect the paper's content?		4			
Does the introduction present the problem clearly?			3		
Are the results/ conclusions justified?			3		
How comprehensive and up-to-date is the subject matter presented?		4			
How adequate is the data presentation?		4			
Are units and terminology used correctly?		4			
Is the number of cases adequate?	5				
Are the experimental methods/ clinical studies adequate?		4			
Is the length appropriate in relation to the content?		4			
Does the reader get new insights from the article?				2	
Please rate the practical significance.		4			
Please rate the accuracy of methods.			3		
Please rate the statistical evaluation and quality control.			3		
Please rate the appropriateness of the figures and tables.			3		
Please rate the appropriateness of the references.			3		
Please evaluate the writing style and use of language.		4			
Please judge the overall scientific quality of the manuscript.			3		
Are the methods used worthy of reproduction in greater deal?	No				
Would you be willing to review a revision of this manuscript?	Yes				

Comments to author: The revised manuscript has led to a major improvement which may allow publication in ISS without further changes.
